# Lowering the quantification limit of the Qubit^TM^ RNA HS Assay using RNA spike-in

**DOI:** 10.1186/s12867-015-0039-3

**Published:** 2015-05-06

**Authors:** Xin Li, Iddo Z Ben-Dov, Maurizio Mauro, Zev Williams

**Affiliations:** Department of Obstetrics & Gynecology and Women’s Health, Albert Einstein College of Medicine, 10461 Bronx, NY USA; Department of Genetics, Albert Einstein College of Medicine, 10461 Bronx, NY USA; Nephrology and Hypertension, Hadassah – Hebrew University Medical Center, 91120 Jerusalem, Israel

**Keywords:** Lower quantification limit, Minimum RNA concentration, Plasma RNA, Qubit™ RNA HS Assay, RNA quantification, RNA spike-in

## Abstract

**Background:**

RNA quantification is often a prerequisite for most RNA analyses such as RNA sequencing. However, the relatively low sensitivity and large sample consumption of traditional RNA quantification methods such as UV spectrophotometry and even the much more sensitive fluorescence-based RNA quantification assays, such as the Qubit™ RNA HS Assay, are often inadequate for measuring minute levels of RNA isolated from limited cell and tissue samples and biofluids. Thus, there is a pressing need for a more sensitive method to reliably and robustly detect trace levels of RNA without interference from DNA.

**Methods:**

To improve the quantification limit of the Qubit™ RNA HS Assay, we spiked-in a known quantity of RNA to achieve the minimum reading required by the assay. Samples containing trace amounts of RNA were then added to the spike-in and measured as a reading increase over RNA spike-in baseline. We determined the accuracy and precision of reading increases between 1 and 20 pg/μL as well as RNA-specificity in this range, and compared to those of RiboGreen®, another sensitive fluorescence-based RNA quantification assay. We then applied Qubit™ Assay with RNA spike-in to quantify plasma RNA samples.

**Results:**

RNA spike-in improved the quantification limit of the Qubit™ RNA HS Assay 5-fold, from 25 pg/μL down to 5 pg/μL while maintaining high specificity to RNA. This enabled quantification of RNA with original concentration as low as 55.6 pg/μL compared to 250 pg/μL for the standard assay and decreased sample consumption from 5 to 1 ng. Plasma RNA samples that were not measurable by the Qubit™ RNA HS Assay were measurable by our modified method.

**Conclusions:**

The Qubit™ RNA HS Assay with RNA spike-in is able to quantify RNA with high specificity at 5-fold lower concentration and uses 5-fold less sample quantity than the standard Qubit™ Assay.

**Electronic supplementary material:**

The online version of this article (doi:10.1186/s12867-015-0039-3) contains supplementary material, which is available to authorized users.

## Background

Recent studies utilizing trace amounts of RNA present in biospecimens such as biofluids, single cells and minute clinical samples have revealed their novel functions and biomedical potentials [1-14]. RNA quantification is an important and necessary step prior to most RNA analyses. However, it can be very challenging to quantify RNA present in the pg/μL ranges found in biofluids and minute cell and tissue samples [6]. After purification using most commercial RNA isolation kits, the concentrations of purified plasma RNA samples are often less than 200 pg/μL. UV spectrophotometry commonly used for nucleic acid quantification has a lower quantification limit around 4 ng/μL, and is therefore not suitable for measuring RNA samples with such low concentrations [15-17].

An alternative approach is fluorescence-based RNA quantification that utilizes the fluorescent property of nucleic acid binding dyes. Unbound dyes are nearly non-fluorescent, but upon binding to nucleic acid, the complex exhibits a large increase in fluorescence, thereby greatly amplifying nucleic acid signal for detection at concentrations much lower than that required by UV spectrophotometry [15,16,18-21]. An example of fluorescence-based RNA quantification methods is the Qubit™ RNA HS Assay (Life Technologies, Thermo Fisher Scientific Inc.).

The Qubit™ RNA HS Assay is highly selective for RNA over DNA [22] and provides a minimum “reading” (RNA concentration in the Qubit™ working solution) of 25 pg/μL with high confidence (deviation from ideal < 20%). Up to 20 μL of RNA sample can be added in a 200 μL Qubit™ Assay, and therefore RNA samples with a minimum starting concentration of 250 pg/μL can be accurately quantified. However, this minimum concentration is still relatively high compared to levels of RNA found in certain biological specimens. Moreover, the assay consumes a minimum of 5 ng of RNA sample, which may leave insufficient RNA for downstream applications. Thus, these detection limitations to the Qubit™ Assay can hinder the analysis and application of some biological samples with extremely low RNA quantities.

Here we used an RNA spike-in to set a baseline reading of the Qubit™ Assay and measured RNA sample as an increase over RNA spike-in. This method was validated to accurately measure RNA at lower concentrations and require less sample compared to standard Qubit™. We tested the utility of this spike-in approach by measuring plasma RNA samples that fell below the detection limit of the standard Qubit™ Assay. We named the modified assay the Spike-in Qubit™ RNA HS Assay because this optimization takes advantage of an RNA spike-in.

## Methods

### Validation of the Spike-in Qubit™ RNA HS assay

The Qubit™ RNA HS Assay Kit (Life Technologies, Thermo Fisher Scientific Inc.), Qubit™ 2.0 Fluorometer (Life Technologies, Thermo Fisher Scientific Inc.) and Axygen PCR-05-C tubes (Axygen) were used for all measurements. The Qubit™ working solution was made according to manufacturer’s instructions. We added 180 μL of working solution to each assay tube, up to 20 μL of RNA, and water to bring the final volume to 200 μL. 10 μL of the Qubit™ RNA Standard solutions were used for standard tubes. “RNA spike-in” was made by diluting the Qubit™ RNA Standard #2 (10 ng/μL rRNA) included in the Qubit™ RNA HS Assay kit to 2.5 ng/μL and 2 μL was added into each tube. 18 μL of water was added into one tube for RNA spike-in alone reading. “RNA sample” was made by diluting the Qubit™ RNA Standard #2 to 250 pg/μL and increasing volumes of RNA sample were added into remaining tubes to create expected reading increases of 1, 2, 3, 4, 5, 7.5, 15 and 20 pg/μL over RNA spike-in alone. Assay tubes were vortexed for 2–3 s, centrifuged briefly (~5 s), and then incubated at room temperature for 2 min to allow the assay to reach optimal fluorescence before measuring with the Qubit™ Fluorometer. Each tube was measured three times to obtain the average reading. Reading increase was calculated by subtracting RNA spike-in reading from that of spike-in plus sample RNA reading. The experiment was repeated four times using independently prepared RNA spike-in, RNA sample, and working solution. In addition, the total RNA from human trophoblast cells was used as “RNA sample” and tested as described above for a total of four independent experiments.

### Comparison between the Spike-in Qubit™ RNA HS Assay and Quant-iT™ RiboGreen® RNA Assay

Based on the original concentration measured by NanoDrop® ND-1000 (Thermo Fisher Scientific Inc.), the trophoblast total RNA was diluted to 60 pg/μL in water. A mixture of RNA and DNA (60 pg/μL each) was prepared by diluting in water the trophoblast total RNA and the Qubit™ DNA Standard #2 (10 ng/μL DNA) included in the Qubit™ DNA HS Assay kit. 18 μL of each sample was measured by the Spike-in Qubit™ as described above and by the Quant-iT™ RiboGreen® RNA Assay (Life Technologies, Thermo Fisher Scientific Inc.) following manufacturer’s instructions using a BioTek Synergy™ 4 Multi-Mode Microplate Reader (BioTek). Four independent repeats were performed with each method.

### Quantification of plasma RNA samples

The standard Qubit™ RNA HS Assay was performed according to manufacturer’s instructions. The Spike-in Qubit™ RNA HS Assay was performed as described above. For the Spike-in Qubit™ Assay, RNA sample concentration was calculated as: [Sample] = reading increase (pg/μL) x assay volume (μL) ÷ sample volume for Spike-in Qubit™ (μL). Three independent measurements using separately prepared RNA spike-in and Qubit™ reagents were made for each sample. A detailed step-by-step protocol for the Spike-in Qubit™ RNA HS Assay is provided in the Supplementary Methods.

### Sample preparation and RNA isolation

After obtaining informed consent, 8.5 mL of peripheral blood samples were collected into Vacutainer™ tubes containing acid citrate dextrose solution A (BD) and immediately inverted eight times to mix anticoagulant additive with blood. Blood samples were centrifuged at 1,900 g for 10 min at 4°C in a swinging-bucket centrifuge (Eppendorf) and plasma was aspirated using disposable transfer pipets (VWR), aliquoted into 2 mL polypropylene microcentrifuge tubes (Sarstedt) and centrifuged at 16,050 g for 5 min at 4°C in a benchtop centrifuge (Eppendorf). The final plasma was pooled and mixed in 15 mL tubes (Falcon), then aliquoted into 1.5 mL DNA LoBind tubes (Eppendorf), snap frozen in liquid nitrogen and stored at −80°C. Plasma RNA isolation was performed using three commercial RNA isolation kits: miRCURY™ RNA Isolation Kit – Biofluids (Exiqon), mirVana™ PARIS™ Kit (Life Technologies, Thermo Fisher Scientific Inc.) and miRNeasy Micro Kit (Qiagen) following manufacturer recommended protocols. The miRCURY™ kit is designed to isolate RNA shorter than 1000 nucleotides (nt) and the mirVana™ and miRNeasy kits isolate total RNA.

After informed consent was obtained, tissues were collected from manual vacuum aspiration. To isolate trophoblast cells, chorionic villi were identified and floated in DPBS supplemented with 10 mg/ml Gentamycin, minced with sterile scalpel blades, placed in 35 ×10 mm dishes and incubated with 3.3 mg/ml Collagenase (Sigma) for about 2 hours at 37°C in the cell incubator. Cells were further washed twice with Amniomax complete medium (Amionax basal medium plus F100 supplement; GIBCO) and cultured in Amniomax complete medium for 7 days. RNA from trophoblast cells was extracted with All Prep® DNA/RNA/Protein mini kit (Qiagen) according to the manufacturer’s protocol. All aspects of these studies were reviewed and approved by the Institutional Review Board at Albert Einstein College of Medicine.

### Data analyses

Data analyses were performed using Excel® for Mac 2011 (Microsoft) and GraphPad Prism 6 (GraphPad Software). Relative error (RE) was calculated as RE = (average measured reading − expected reading) ÷ expected reading. Coefficient of variation (CV) was calculated as CV = standard deviation ÷ average measured reading. Deviation from ideal was calculated as the sum of the absolute value of RE and CV. A two-way ANOVA interaction analysis was performed to determine whether the differences between the measurements of the 60 pg/μL RNA sample and those of the mixed RNA and DNA sample (60 pg/μL each) were consistent for the Spike-in Qubit™ RNA HS Assay and the Quant-iT™ RiboGreen® RNA Assay.

## Results and discussion

**Figure 1:**
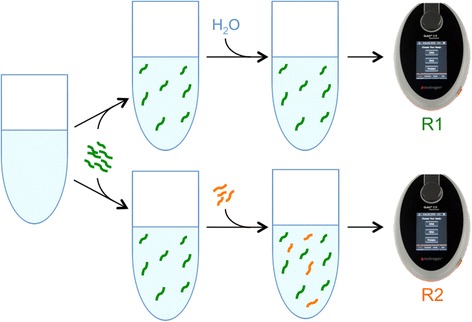
Schematic diagram of the Spike-in Qubit™ RNA HS Assay. RNA spike-in (green) was added to reach the lower quantification limit of Qubit™. Then nuclease-free water or RNA sample (orange) was added for Qubit™ measurement. R1 is the reading for RNA spike-in alone and R2 for RNA spike-in plus RNA sample. The reading for RNA sample is (R2 - R1) and RNA sample concentration is calculated as [sample] = (R2 – R1) (pg/μL) × assay volume (μL) ÷ sample volume for the assay (μL).

The lower reading limit of the Qubit™ RNA HS assay for accurate quantification is 25 pg/μL (deviation from ideal < 20%). In order to quantify samples that fall below this limit, prior to adding the RNA sample that was to be measured, we added 5 ng of RNA spike-in (2.5 ng/μL Qubit™ RNA standard #2) into a 200 μL Qubit™ assay to generate an expected baseline reading of 25 pg/μL. Then, the RNA sample was added to the baseline and the increase over baseline would correspond to the reading of RNA sample (Figure 1).

**Figure 2:**
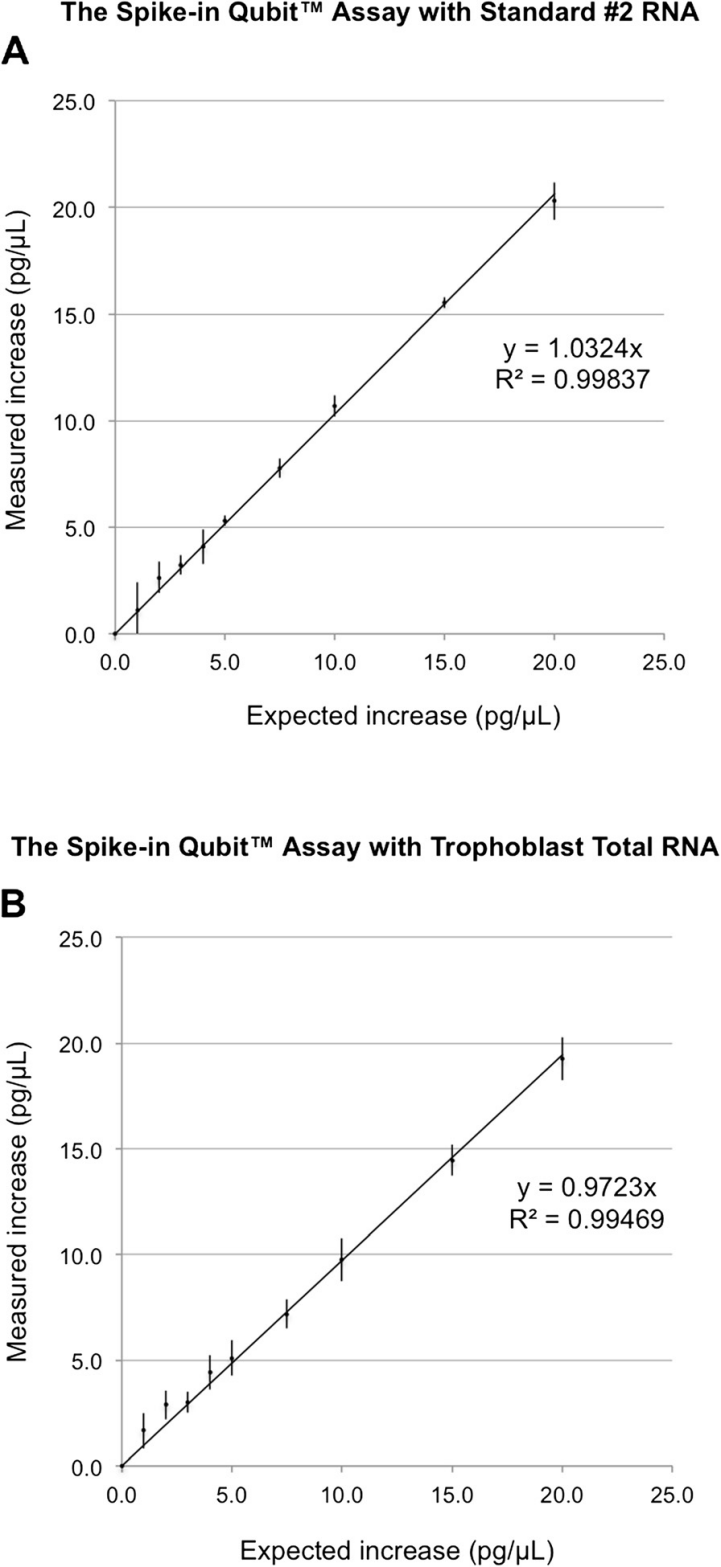
Reading increases between 1 and 20 pg/μL show a strong linear correlation in the Spike-in Qubit™ RNA Assay. RNA spike-in alone or with increasing amounts of a 250 pg/μL Qubit™ RNA Standard #2 sample **(A)** or a 250 pg/μL trophoblast total RNA sample **(B)** was measured by the Qubit™ Assay. Reading increases over RNA spike-in were plotted against expected reading increases. Regression line equation, coefficient of determination (R^2^) and error bars indicating standard deviation are shown. N = 4 independent repeats.

To evaluate the ability of our Spike-in Qubit™ Assay to measure increases in the range of 1 to 20 pg/μL, an RNA sample (250 pg/μL Qubit™ RNA standard #2) was added at increasing volumes into Qubit™ assay tubes containing RNA spike-in to create expected reading increases ranging from 1 to 20 pg/μL. As shown in Figure 2A, the Spike-in Qubit™ Assay achieved optimal linear regression with slope of 1.0324, and R^2^ of 0.99837 after four independent experiments (Additional file 2: Table S1). To represent a typical RNA sample that might introduce additional influences on fluorometric measurement, the same experiment was repeated with a 250 pg/μL human trophoblast total RNA sample. Strong linear correlation was also evident with slope of 0.9723 and R^2^ of 0.99469 (Figure 2B) after four independent experiments (Additional file 2: Table S2).

We assessed the accuracy and precision of the Spike-in Qubit™ Assay for all tested reading increases in order to determine the lower quantification limit that passed accuracy and precision requirements. Accuracy is inversely correlated to the relative error (RE) and precision is inversely correlated to the coefficient of variation (CV). The Qubit™ RNA HS Assay used “deviation from ideal”, the combined value of the absolute value of RE and CV, to evaluate the precision and accuracy and set deviation from ideal of <20% for its lower quantification limit in the core quantification range. Using the same criterion, we determined that Spike-in Qubit™ Assay achieved a lower quantification limit of 5 pg/μL for both Qubit™ Standard #2 RNA and trophoblast total RNA, which is 80% less than that of the standard Qubit™ Assay (25 pg/μL) (Tables 1 and 2). This new lower quantification limit allowed quantification of RNA samples with original concentrations as low as 55.6 pg/μL and reduced minimal sample consumption from 5 ng to 1 ng (Table 3).

**Table 1:** **The Spike-in Qubit™ RNA HS Assay achieves 5 pg/μL lower quantification limit for the Qubit™ RNA standard #2**

**Expected increase (pg/μL)**	**Ave. measured increase ± SD (pg/μL)**	**Relative error**	**Coefficient of variation**	**Deviation from ideal**
1	1.1 ± 1.3	12.5%	114.6%	127.1%
2	2.7 ± 0.7	32.5%	27.6%	60.1%
3	3.2 ± 0.4	7.5%	13.6%	21.1%
4	4.1 ± 0.8	2.5%	19.9%	22.4%
5	5.3 ± 0.2	6.3%	4.6%	10.9%
7.5	7.8 ± 0.5	3.6%	5.8%	9.4%
10	10.7 ± 0.5	6.9%	4.7%	11.6%
15	15.5 ± 0.3	3.4%	1.7%	5.1%
20	20.3 ± 0.9	1.6%	4.4%	6.0%

A 250 pg/μL Qubit™ RNA Standard #2 RNA sample was used to assess the accuracy and precision of reading increases between 1 and 20 pg/μL using the Spike-in Qubit™. Average measured reading increase ± standard deviation (SD), relative error, coefficient of variation, and deviation from ideal expressed in percent are listed in the table. N = 4 independent repeats.

**Table 2:** **The Spike-in Qubit™ RNA HS Assay achieves 5 pg/μL lower quantification limit for trophoblast total RNA**

**Expected increase (pg/μL)**	**Ave. measured increase ± SD (pg/μL)**	**Relative error**	**Coefficient of variation**	**Deviation from ideal**
1	1.7 ± 0.8	65.8%	50.6%	116.5%
2	2.9 ± 0.7	44.2%	23.9%	68.0%
3	3.0 ± 0.5	0.6%	16.7%	17.3%
4	4.4 ± 0.8	10.6%	18.4%	29.1%
5	5.1 ± 0.8	2.2%	16.3%	18.5%
7.5	7.2 ± 0.7	**−**4.1%	9.6%	13.7%
10	9.7 ± 1.0	**−**2.6%	10.5%	13.0%
15	14.5 ± 0.8	**−**3.6%	5.2%	8.8%
20	19.3 ± 1.0	**−**3.7%	5.3%	9.0%

A 250 pg/μL trophoblast total RNA sample was used to assess the accuracy and precision of reading increases between 1 and 20 pg/μL using the Spike-in Qubit™. Average measured reading increase ± standard deviation (SD), relative error, coefficient of variation, and deviation from ideal expressed in percent are listed in the table. N = 4 independent repeats.

**Table 3:** **The spike-in Qubit™ RNA HS Assay reduces minimal RNA concentration and sample consumption**

	**Qubit** ^**TM**^	**Spike-in Qubit** ^**TM**^
Lower Quantification Limit (pg/μL)	25	5
Max. Sample Volume (μL)	20	18*
Min. Sample Concentration (pg/μL)	250	55.6
Min. Sample Quantity (ng)	5	1

The lower quantification limit and maximum sample volume for the Qubit™ Assay and Spike-in Qubit™ Assay and their corresponding minimal sample concentration and quantity are listed in the table. *2 μL RNA Spike-in is added into each assay tube, leaving maximally 18 μL for RNA sample in a 200 μL assay.

We also cross-compared the precision and accuracy of the Spike-in Qubit™ Assay with those of the Quant-iT™ RiboGreen® RNA Assay that has a lower quantification limit of 1 pg/μL. A 60 pg/μL trophoblast total RNA sample was measured in four independent repeats with both methods (Additional file 2: Table S3 and S4). As shown in Table 4, the RNA concentration measured by the Spike-in Qubit™ was 63.6 ± 3.4 pg/μL and 53.4 ± 1.3 pg/μL by the Quant-iT™. Both methods achieved good precision with CVs of 5.4% and 2.5% for the Spike-in Qubit™ and the Quant-iT™, respectively. In contrast to the Quant-iT™ RiboGreen® RNA Assay and most other methods including UV spectrometer, the Qubit™ RNA Assay is reported to be selective to RNA over DNA [15,16,18,22]. DNA at 8 times higher concentration of the lower quantification limit (25 pg/μL) is not detectable in the Qubit™ RNA Assay and an equal mixture of DNA and RNA up to 200 pg/μL each in the assay does not affect the reading of RNA [22]. To test if the Spike-in Qubit™ maintains RNA specificity at low readings, we mixed the 60 pg/μL RNA with 60 pg/μL DNA. The reading for the mixture was 64.2 ± 6.2 pg/μL, similar to the 60 pg/μL RNA concentration measured by the Spike-in Qubit™. In contrast, the presence of DNA significantly increased the quantification value by the Quant-iT™ from 53.4 ± 1.3 pg/μL for the RNA sample to 190.1 ± 6.1 pg/μL for the RNA plus DNA sample. A two-way ANOVA analysis determined that the differences in the measurements of the RNA sample and those of the mixed RNA and DNA sample were significantly different between the Spike-in Qubit™ RNA HS Assay and the Quant-iT™ RiboGreen® RNA Assay (P < 0.0001).

**Table 4:** **The Spike-in Qubit™ RNA HS Assay maintains high precision and RNA specificity in the extended lower reading range**

	**Spike-in Qubit** ^**TM**^		**Quant-it** ^**TM**^	
**Sample**	**Reading ± SD (pg/μL)**	**[RNA] ± SD (pg/μL)**	**Reading ± SD (pg/μL)**	**[RNA] ± SD (pg/μL)**
60 pg/μL RNA	5.7 ± 0.3	63.6 ± 3.4	4.8 ± 0.1	53.4 ± 1.3
60 pg/μL RNA + 60 pg/μL DNA	5.8 ± 0.6	64.2 ± 6.2	17.1 ± 0.6	190.1 ± 6.1

A 60 pg/μL RNA sample and a mixture of 60 pg/μL RNA and 60 pg/μL DNA were measured by the Spike-in Qubit™ and the Quant-iT™ RiboGreen® Assays. Average reading ± standard deviation (SD) and corresponding RNA concentration ± SD are listed in the table. N = 4 independent repeats.

RNA samples purified from serum or plasma are present at low concentration (~30 ng per 1 mL of plasma), making their quantification challenging [6,23,24]. To determine whether our newly validated Spike-in Qubit™ Assay would enable accurate measurements from these samples which would normally be at too low of a concentration to be measured with standard Qubit™, we used both assays to measure plasma RNA samples purified using three commercial RNA isolation kits. All three samples fell below the detection limit of the standard Qubit™ and therefore their concentrations could not be determined (Table 5). In contrast, The Spike-in Qubit™ Assay achieved quantification for all samples while consuming 25-50% less samples than the standard Qubit™ (Table 5).

**Table 5:** **The spike-in Qubit™ RNA HS Assay enables quantification of RNA samples purified from plasma**

	**Qubit™**			**Spike-in Qubit™**			
**Kit**	**Sample vol. (μL)**	**Reading (pg/μL)**	**[RNA] (pg/μL)**	**Sample Vol. (μL)**	**Reading ± SD (pg/μL)**	**[RNA] ± SD (pg/μL)**	**CV**
1	20	<20	N.D.	10	5.3 ± 0.7	106.0 ± 14.0	13.2%
2	20	< 20	N.D.	15	13.2 ± 1.8	176.4 ± 23.4	13.3%
3	20	< 20	N.D.	15	6.0 ± 0.3	80.0 ± 3.5	4.4%

Plasma RNA samples purified using the three kits listed in the Methods were quantified by the Qubit™ and the Spike-in Qubit™ Assays. Sample volume used for quantification, Qubit™ reading or Spike-in Qubit™ reading increase ± standard deviation (SD) and corresponding RNA concentration ± SD and coefficient of variation (CV) are listed in the table. “ < 20” indicates the reading is below the Qubit™ detection limit and therefore sample RNA concentration could not be determined (N.D.) For the Spike-in Qubit™, RNA sample concentration was calculated as described in the Methods. N = 3 independent repeats.

We speculate that the Spike-in Qubit™ approach may work because the readings of RNA spike-in alone and with additional RNA samples fall into the linear and high-precision quantification range of the Qubit™. It ensures that the reading increase over RNA spike-in baseline is of high precision and linear therefore of high accuracy. There are limitations to the Spike-in Qubit™ RNA HS Assay. First, it requires extra steps to prepare and add RNA spike-in. However, these steps only consist of diluting the Qubit™ RNA Standard #2 (that comes pre-made in the kit) and adding it into a master mix. Therefore, the time and risk of introducing error are minimal. In addition, because an excess amount of Qubit™ RNA Standard #2 is provided in the Qubit™ Assay kit, there is no need to purchase additional reagents. As the validation for reading increases between 1 and 5 pg/μL was performed by adding RNA samples in 0.8 to 4 μL volumes, Pipetting of small volumes could have contributed to variation in measurements due to pipetting error.

## Conclusions

The Spike-in Qubit™ RNA HS Assay reported here achieved accurate and precise RNA quantification at a new lower quantification limit of 5 pg/μL, 5-fold lower than that of the standard Qubit™, while maintaining the RNA specificity of the original assay. This improvement lowers minimal RNA concentration measurable from 250 pg/μL to 55.6 pg/μL and reduces minimal RNA consumption from 5 ng to 1 ng. As demonstrated in the successful quantification of plasma RNA samples, the Spike-in Qubit™ RNA HS can be readily used to quantify RNA samples having low concentrations and limited quantities.

## Additional files

Additional file 1:
**The Spike-in Qubit™ RNA HS Assay Protocol.**
Additional file 2: Table S1. Four independent Spike-in Qubit™ measurements of reading increases between 1 and 20 pg/μL using a 250 pg/μL Qubit™ Standard #2 RNA sample. **Table S2.** Four independent Spike-in Qubit™ measurements of reading increases between 1 and 20 pg/μL using a 250 pg/μL trophoblast total RNA sample. **Table S3.** Four independent Spike-in Qubit™ measurements of a 60 pg/μL trophoblast total RNA sample or a mixure of trophoblast total RNA and Qubit™ Standard #2 DNA (60 pg/μL each). **Table S4.** Four independent Quant-iT™ RiboGreen® measurements of a 60 pg/μL trophoblast total RNA sample or a mixure of trophoblast total RNA and Qubit™ Standard #2 DNA (60 pg/μL each). **Table S5.** Three independent Spike-in Qubit™ measurements of plasma RNA samples.

## Abbreviations

CV: Coefficient of variation
RE: Relative error
SD: Standard deviation
