# Supplementary material for: Lowering the quantification limit of the QubitTM RNA HS Assay using RNA spike-in
Source: BMC Mol Biol. 2015 May 6;16:9. doi: 10.1186/s12867-015-0039-3 (PMC4431604; doi:10.1186/s12867-015-0039-3)
Supplement: Additional file 1: — The Spike-in Qubit™ RNA HS Assay Protocol. [file 12867_2015_39_MOESM1_ESM.docx]

**Additional file 1**

**The Spike-in Qubit™ RNA HS Assay Protocol**

**Step 1**: Determine total number of assay tubes, including 2 standards, 1 RNA Spike-in alone and “n” RNA samples. Label tubes.

**Step 2**: Prepare sufficient amount of 2.5 ng/μL RNA spike-in for 1 RNA Spike-in alone and “n” RNA samples, by making a 4-fold dilution of the Qubit™ RNA Standard #2 (10 ng/μL) with RNase and DNase-free water.

**Step 3**: Prepare sufficient Qubit™ working solution for all tubes using 1 μL of Qubit™ RNA reagent and 199 μL of Qubit™ RNA buffer per tube. Mix well.

**Step 4**: Prepare Standard tubes by adding 180 μL working solution, 10 μL water and 10 μL Qubit™ Standard #1 or #2 solution into each tube.

**Step 5**: Add RNA spike-in into working solution to make a master mix sufficient for 1 RNA Spike-in alone and “n” RNA samples, by using 2 μL of RNA spike-in and 180 μL of the Qubit™ working solution per tube. Mix well.

**Step 6**: Aliquot 182 μL of the master mix into each tube. Add 18 μL of water into the RNA spike-in alone tube. Add RNA samples and water for 18 μL total into RNA sample tubes.

**Step 6**: Mix solution by vortexing for 2 - 3 seconds and centrifuge for ~5 seconds to collect solution. To allow the Qubit™ assay to reach optimal fluorescence, incubate tubes for 2 minutes at room temperature (RT). After this incubation period, the fluorescence signal is stable for 3 hours at RT.

**Step 7**: Measure the RNA spike-in alone tube (Read1) and RNA sample tubes (Read2) with the Qubit™ 2.0 Fluorometer and calculate sample concentration as [Sample] = (Read2 – Read1) (pg/μL) × 200 (μL) ÷ volume of sample added (μL).
